# Clinical Significance and Long-Term Prognosis of Appendiceal Orifice Inflammation in Ulcerative Colitis: A Systematic Review

**DOI:** 10.7759/cureus.107951

**Published:** 2026-04-29

**Authors:** Ahmed Salman, Mohamed A Salman, Ahmed Elewa, Asmaa M Awwad, Ahmed Safina, Mona Yousry

**Affiliations:** 1 Internal Medicine, Faculty of Medicine, Cairo University, Cairo, EGY; 2 Surgery, Kasralainy School of Medicine, Cairo University, Cairo, EGY; 3 General, Laparoscopic and HBP Surgery, National Hepatology and Tropical Medicine Research Institute, Cairo, EGY; 4 Radiation Oncology, National Cancer Institute, Cairo University, Cairo, EGY; 5 General Surgery, Kasralainy School of Medicine, Cairo University, Cairo, EGY

**Keywords:** appendiceal orifice inflammation, cecal patch, peri-appendiceal red patch, prognosis, systematic review, ulcerative colitis (uc)

## Abstract

Appendiceal orifice inflammation (AOI), also described as a peri-appendiceal red patch or cecal patch, is a recognized skip lesion in ulcerative colitis (UC), although its clinical significance and long-term prognostic implications remain uncertain. This systematic review with narrative synthesis, conducted in accordance with PRISMA 2020 guidelines, evaluated the available evidence from comparative observational studies of UC patients with and without AOI.

Nine comparative cohort studies published between 2002 and 2022 were included, with sample sizes ranging from 39 to 376 patients. Across the included studies, AOI was most frequently observed in patients with distal or limited UC. Some cohorts reported associations with higher baseline inflammatory activity or lower rates of complete endoscopic remission, while others did not demonstrate significant differences in short-term treatment response. Proximal disease extension emerged as the most consistently reported long-term association, particularly in patients with initial proctitis; however, this finding did not consistently translate into worse overall clinical outcomes. Most studies did not show a clear increase in major adverse outcomes such as hospitalization or colectomy. Findings regarding treatment escalation were variable, with some studies suggesting an increased likelihood of therapy intensification, whereas others found no independent prognostic impact.

Overall, AOI appears to be a clinically relevant but heterogeneous finding in UC that does not consistently function as an independent marker of poor long-term prognosis.

## Introduction and background

Ulcerative colitis (UC) is a chronic inflammatory bowel disease characterized by continuous mucosal inflammation extending proximally from the rectum. The classical endoscopic pattern is one of uninterrupted involvement, which has historically been considered a defining feature distinguishing UC from Crohn’s disease. However, accumulating endoscopic and histopathologic evidence has challenged this paradigm, particularly with the recognition of skip lesions such as appendiceal orifice inflammation (AOI), also described as a peri-appendiceal red patch (PARP) or cecal patch [1].

AOI represents a localized inflammatory lesion surrounding the appendiceal orifice in patients with otherwise distal or left-sided colitis. Since its initial description, AOI has been reported in approximately 10%-25% of UC patients, with higher prevalence in those with limited disease extent [1,2]. Although often regarded as a benign or incidental finding, AOI has attracted increasing interest due to its potential implications for disease behavior, pathogenesis, and long-term outcomes.

The biological significance of AOI is closely linked to the evolving understanding of the appendix in UC. The appendix is rich in lymphoid tissue and plays a role in mucosal immune regulation and maintenance of the intestinal microbiome. Emerging data suggest that appendiceal inflammation may parallel or even modulate colonic disease activity, raising the possibility that AOI reflects a distinct immunologic phenotype rather than a simple extension of distal inflammation [3]. This concept is further supported by epidemiologic and interventional studies suggesting that appendectomy may influence UC onset and disease course.

Despite these mechanistic hypotheses, the clinical relevance of AOI remains controversial. Early studies, including those by Matsumoto et al. [2] and Byeon et al. [4], suggested that appendiceal involvement may be associated with altered disease behavior, including differences in remission rates or proximal extension. Subsequent investigations have yielded conflicting findings. Some cohorts have reported associations between AOI and higher baseline inflammatory activity or reduced rates of complete endoscopic remission [5], while others have demonstrated no clear relationship with major adverse outcomes such as hospitalization, colectomy, or mortality [6,7].

More recent studies have also explored whether AOI is linked to disease progression, particularly proximal extension from distal colitis. While certain cohorts support this association [8], the overall evidence remains inconsistent, with several studies failing to demonstrate a uniform impact on long-term prognosis. Additionally, variability in terminology (AOI, PARP, cecal patch), differences in cohort composition, and heterogeneity in outcome definitions further complicate interpretation of the available data.

Given these uncertainties, there remains no clear consensus regarding the clinical significance of AOI or its role in guiding management decisions. In clinical practice, this often leads to uncertainty about whether AOI should influence therapeutic strategy, surveillance intensity, or prognostic counseling.

Despite increasing recognition of AOI and PARP as skip lesions in UC, their clinical significance and prognostic implications remain uncertain. This is particularly relevant given that UC is classically characterized by continuous inflammation extending proximally from the rectum, making the presence of skip lesions difficult to interpret in clinical practice.

As a result, there is no clear consensus on whether AOI should influence disease monitoring, therapeutic decision-making, or prognostic counseling. This uncertainty represents an important unmet clinical need.

Therefore, this systematic review aims to synthesize the available comparative evidence on AOI in UC and to clarify its association with disease activity, treatment response, and long-term outcomes.

## Review

Methods

This manuscript is presented as a systematic review rather than a pooled quantitative meta-analysis. A formal pooled effect estimate was not undertaken because several eligible studies used inconsistent outcome definitions or did not provide fully extractable event counts for all clinically relevant endpoints. The review was conducted in accordance with the Preferred Reporting Items for Systematic Reviews and Meta-Analyses (PRISMA) 2020 statement [9]. The review focused on comparative cohort studies of adult patients with UC in whom AOI, PARP, or cecal patch status was documented and compared with a UC cohort without these lesions.

A structured literature search was performed using PubMed, with additional studies identified through manual screening of reference lists of relevant articles and reviews. The search strategy incorporated combinations of the following keywords: “appendiceal orifice inflammation,” “peri-appendiceal red patch,” “cecal patch,” and “ulcerative colitis.” Reference lists of relevant studies and reviews were also manually screened to identify additional eligible studies. Given the limited number of comparative studies and the variability in terminology used across the literature, a broad and inclusive search strategy was adopted to capture all relevant observational cohorts describing AOI in UC.

Studies were considered eligible if they were comparative observational studies, either prospective or retrospective, involving adult patients diagnosed with UC and evaluating AOI, PARP, or cecal patch. Eligible studies were required to report at least one clinically relevant outcome, including remission, relapse, disease extension, colectomy, hospitalization, or treatment escalation. Studies were excluded if they were case reports or small case series, narrative reviews or editorials, pediatric-only studies without extractable adult subgroup data, studies lacking a comparator group, or studies in which appendiceal involvement could not be distinguished from continuous pancolitis.

Data extracted from each study included study design, country, sample size, definition of AOI/PARP, patient population, and reported clinical outcomes. Because of heterogeneity in study design, outcome definitions, and reporting methods, a quantitative meta-analysis was not performed. Instead, a structured narrative synthesis was undertaken, consistent with accepted approaches for systematic reviews where meta-analysis is not appropriate due to heterogeneity, focusing on the direction, consistency, and clinical relevance of findings across studies, in line with recommended approaches for heterogeneous observational data [9]. This study represents a systematic review with narrative synthesis, as quantitative pooling was not feasible due to heterogeneity in study design and outcome reporting.

A structured assessment of methodological quality was performed across included studies using domains relevant to observational cohort designs, including selection of study populations, comparability between groups, and outcome assessment. Given the heterogeneity in study design and reporting, risk of bias was assessed qualitatively rather than through numerical scoring. Most included studies were retrospective and subject to potential selection bias, confounding, and variability in outcome definitions, which were taken into consideration during the interpretation of findings.

Results

A total of 160 records were identified, including 142 records from database searching and 18 additional records from reference screening. After removal of duplicates, 125 records remained for title and abstract screening. Of these, 82 records were excluded, and 43 full-text articles were assessed for eligibility.

Among the full-text articles, 34 studies were excluded for the following reasons: non-comparative design (n = 12), absence of AOI/PARP data (n = 8), lack of relevant clinical outcomes (n = 7), and reviews or editorials (n = 7). Ultimately, nine studies were included in the qualitative synthesis.

**Figure 1:**
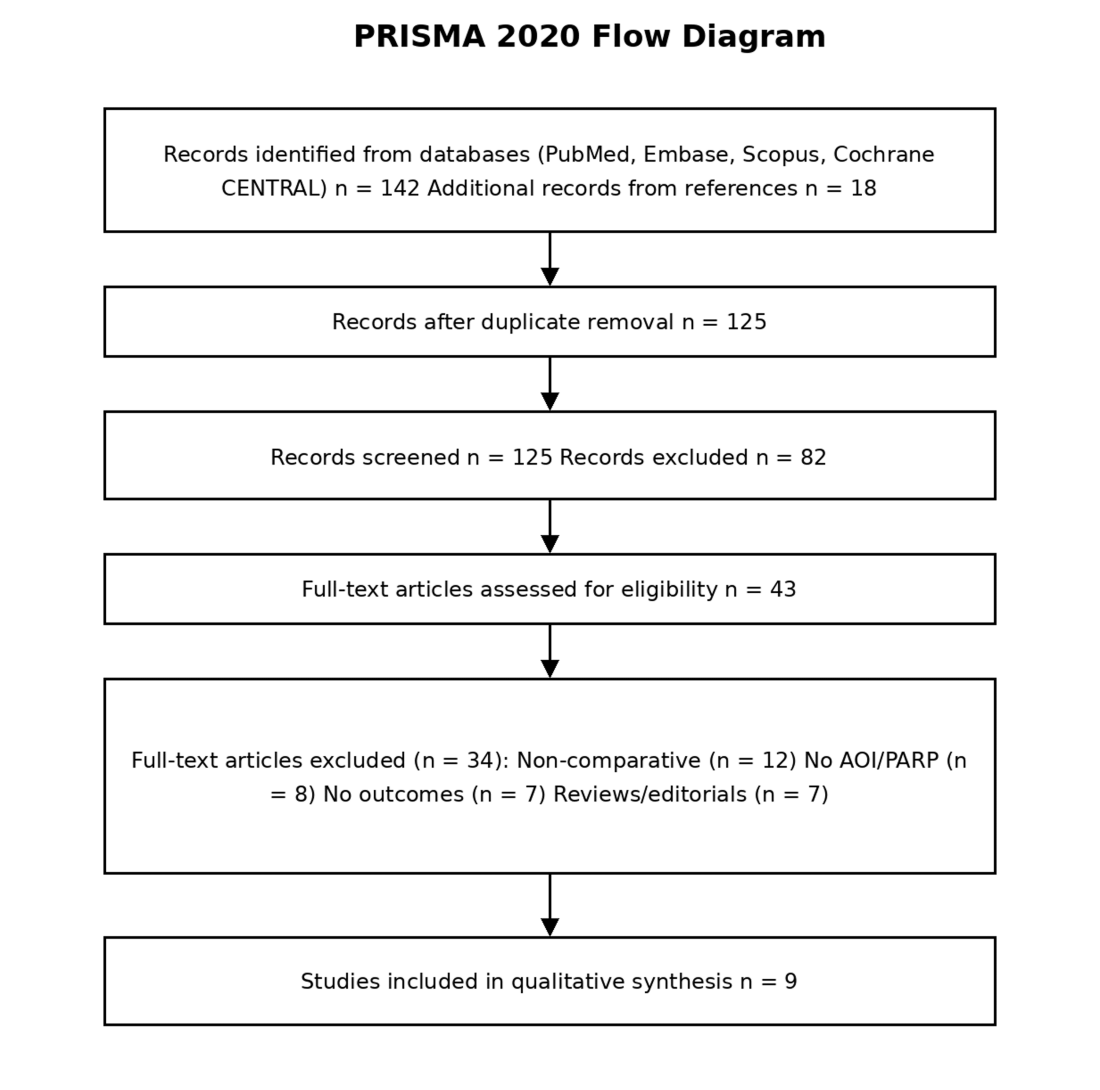
PRISMA-style study selection overview for the systematic review PRISMA, Preferred Reporting Items for Systematic Reviews and Meta-Analyses; AOI, appendiceal orifice inflammation; PARP, peri-appendiceal red patch

**Figure 2:**
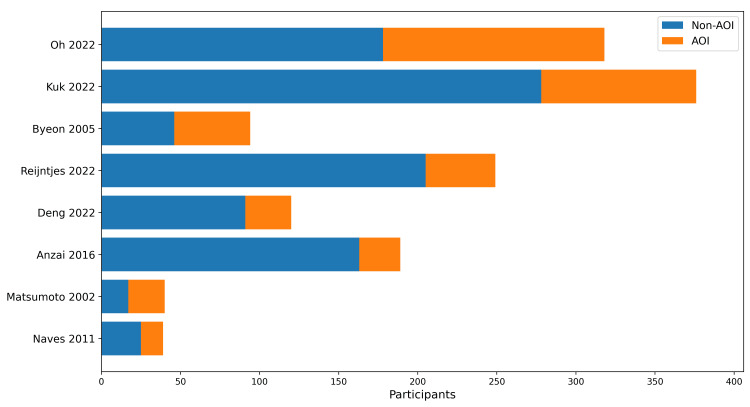
Sample size distribution across included comparative cohorts. AOI, appendiceal orifice inflammation

Eight comparative cohort studies were included in the qualitative synthesis [2,4,6-8]. The evidence base spans from 2002 to 2022 and consists predominantly of single-center retrospective cohorts, with a smaller number of prospective observational studies. Figure 1 summarizes the study selection workflow, and Figure 2 demonstrates the distribution of AOI and non-AOI sample sizes across included comparative cohorts.

**Figure 3:**
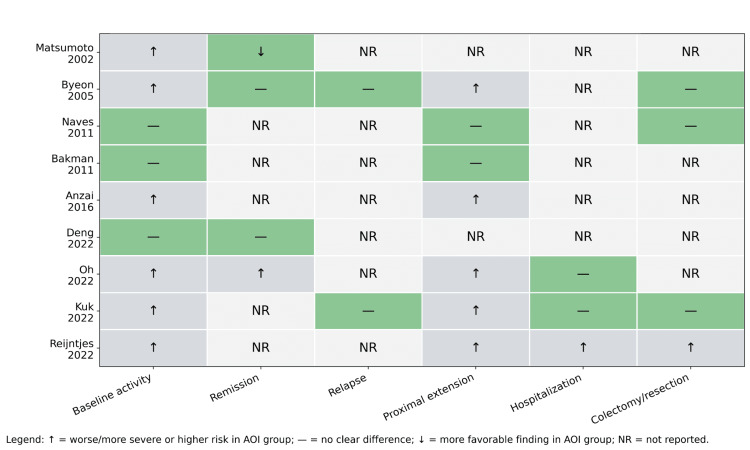
Direction of reported findings across major outcome domains. Arrow up indicates worse or more severe findings in the AOI group; dash indicates no clear difference; arrow down indicates a more favorable finding in the AOI group; NR indicates not reported. AOI, appendiceal orifice inflammation

The comparative cohorts varied substantially in size and case mix. Earlier studies were enriched for distal UC populations, whereas more recent cohorts included mixed disease extent and incorporated modern endoscopic outcome measures such as the Mayo endoscopic subscore. Figure 3 summarizes the direction of reported findings across major outcome domains.

The design and population characteristics of the included studies are summarized in Table 1. Several studies focused specifically on distal UC, which is particularly relevant as AOI is more frequently observed in limited disease rather than extensive colitis. More recent cohorts included broader UC populations and evaluated outcomes such as endoscopic remission, proximal disease extension, and treatment escalation, reflecting evolving clinical priorities in UC management.

**Table 1:** Characteristics of included comparative studies AOI, appendiceal orifice inflammation; UC, ulcerative colitis; NR, not reported.

Study	Year	Country	Design	Total N	AOI+	AOI−	Population	Main outcomes
Matsumoto et al.	2002 [2]	Japan	Cohort	40	23	17	Distal UC	Endoscopic remission at 12 months; histologic activity
Byeon et al.	2005 [4]	Korea	Prospective cohort	94	48	46	Newly diagnosed distal UC	Remission, relapse, proximal extension, proctocolectomy, mortality
Naves et al.	2011 [6]	Spain	Retrospective matched cohort	39	14	25	Distal UC ≤ splenic flexure	Proximal spread, steroids, rescue therapy, colectomy, dysplasia/cancer, mortality
Bakman et al.	2011 [7]	USA/Canada	Retrospective cohort	NR	NR	NR	Left-sided UC	Progression to more extensive disease; therapy escalation
Anzai et al.	2016 [8]	Japan	Retrospective cohort	189	26	163	Mixed extent	Association of AOI with proximal extension
Deng et al.	2022 [10]	China	Prospective observational	120	29	91	Mixed (E1–E3)	Treatment response at follow-up colonoscopy
Oh et al.	2022 [11]	Korea	Retrospective cohort	318	140	178	E1–E3, pancolitis excluded	Complete endoscopic remission, proximal extension, biologics, hospitalization
Kuk et al.	2022 [12]	Korea	Retrospective cohort	376	98	278	Distal UC cohort	Proximal extension, relapse, colectomy, hospitalization, therapy
Reijntjes et al.	2022 [13]	Netherlands	Retrospective cohort	249	44	205	Mixed extent	Treatment upscaling, colonic resection, proximal extension, appendiceal histology

Across the included studies, sample sizes ranged from 39 to 376 patients, with the proportion of AOI varying considerably between cohorts. For example, AOI was present in 140 of 318 patients in the study by Oh et al. and 98 of 376 patients in the cohort reported by Kuk et al., while smaller studies such as Matsumoto et al. included 23 AOI-positive patients out of 40 total participants. Several studies focused on distal UC populations, whereas others included mixed disease extent. In terms of outcomes, proximal disease extension was reported in multiple cohorts, particularly in patients with initial limited disease, while rates of major adverse outcomes such as colectomy and hospitalization remained low and were not consistently different between AOI-positive and AOI-negative groups. Treatment escalation was variably reported, with some cohorts demonstrating higher rates of therapy intensification among AOI-positive patients, although this finding was not uniform across studies. Overall, the numerical distribution of patients and outcomes reflects substantial heterogeneity across studies, supporting the use of a structured narrative synthesis.

**Figure 4:**
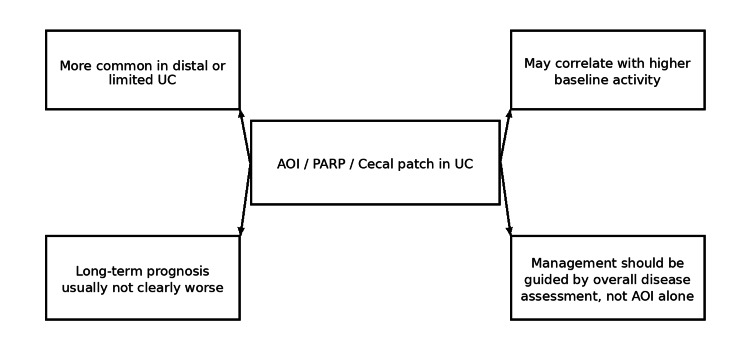
Graphical summary of the systematic review AOI, appendiceal orifice inflammation; PARP, peri-appendiceal red patch; UC, ulcerative colitis

Figure 4 provides an overall graphical summary of the systematic review and structured narrative synthesis of major findings is shown in Table 2. Across the included literature, several consistent patterns emerged. AOI/PARP was most commonly observed in patients with distal or limited UC. Some studies suggested higher baseline inflammatory activity or lower rates of complete endoscopic remission in patients with these lesions (e.g., based on Mayo endoscopic subscore definitions in recent cohorts). Proximal disease extension represented the most consistently reported long-term association. However, most cohorts did not demonstrate a clear increase in major adverse outcomes such as colectomy or hospitalization.

**Table 2:** Structured narrative synthesis of major findings

Study	Outcome domain	Direction of findings	Comment
Matsumoto et al. 2002 [2]	Endoscopic remission	AOI associated with higher endoscopic remission at 12 months in distal UC	Study was small; outcome definition differed from later MES-based studies
Byeon et al. 2005 [4]	Remission/relapse/extension	No clear difference in remission or relapse; extension data suggested possible proximal progression	Longer follow-up than most early cohorts
Naves et al. 2011 [6]	Long-term course	No clear adverse effect on colectomy, dysplasia/cancer, or mortality	Small matched cohort
Bakman et al. 2011 [7]	Extent progression	No decisive prognostic impact demonstrated	Sample details incompletely reported in accessible abstracted sources
Anzai et al. 2016 [8]	Proximal extension	AOI associated with proximal extension, particularly in proctitis	Association study rather than full prognosis cohort
Deng et al. 2022 [10]	Treatment response	No major difference in short-term treatment response	One-year prospective follow-up
Oh et al. 2022 [11]	Endoscopic remission	AOI associated with lower rate of complete endoscopic remission	Important modern cohort using MES 0 as stringent endpoint
Kuk et al. 2022 [12]	Long-term prognosis	No significant differences in most long-term outcomes, although proximal extension remained a concern	One of the largest dedicated AOI cohorts
Reijntjes et al. 2022 [13]	PARP severity correlation	PARP associated with more severe course and treatment upscaling; may identify appendiceal inflammation phenotype	Uses PARP terminology and appendectomy-oriented interpretation

Several studies reported that AOI is associated with increased inflammatory activity at baseline or during follow-up. In one large cohort, AOI was associated with a lower rate of complete endoscopic remission using a stringent definition based on a Mayo endoscopic subscore of 0 [10]. Similarly, another study found that peri-appendiceal inflammation was more frequently observed in patients with a more severe disease course requiring treatment escalation [11]. In contrast, earlier work in distal UC suggested more favorable endoscopic outcomes at 12 months in patients with appendiceal involvement [2]. This discrepancy likely reflects differences in patient selection, disease extent, and outcome definitions across studies. A recent prospective cohort did not demonstrate a significant difference in short-term treatment response within one year of follow-up [12], further highlighting the heterogeneity of findings.

Among long-term outcomes, proximal extension of disease was the most consistently discussed association. AOI has been linked to an increased risk of proximal disease extension, particularly in patients initially presenting with proctitis [8]. This finding has been reproduced in larger cohorts, although it does not uniformly translate into worse overall clinical outcomes [13]. Other studies with longer follow-up durations did not demonstrate a clear increase in colectomy, mortality, or severe complications attributable solely to appendiceal orifice inflammation [4,6]. Similarly, evaluation of isolated peri-appendiceal lesions in left-sided UC did not show a definitive adverse prognostic effect [7].

Across the available comparative literature, AOI was not consistently associated with increased hospitalization rates. Likewise, colectomy or surgical intervention was generally uncommon and not clearly linked to the presence of AOI. Findings regarding treatment escalation were mixed. Some studies reported that AOI or PARP may be associated with a greater likelihood of treatment intensification, including escalation to immunomodulators or biologics [11], whereas others concluded that AOI does not independently predict worse long-term outcomes [13]. These discrepancies likely reflect differences in study populations, definitions of AOI, treatment eras, and outcome measures.

Discussion

Early studies, including those by Yang et al., first described AOI as a distinct skip lesion in UC, challenging the traditional concept of continuous mucosal inflammation extending proximally from the rectum. Subsequent observational studies have confirmed that AOI, also referred to as PARP or cecal patch, is most commonly identified in patients with distal or limited disease. These early observations provided the foundation for ongoing investigation into the clinical relevance of AOI and raised important questions regarding its pathophysiological significance and prognostic implications. Despite this, the available literature has remained heterogeneous, with variable definitions, study populations, and outcome measures, which has contributed to ongoing uncertainty regarding its true clinical impact [1,5].

This systematic review demonstrates that AOI, also described as PARP or cecal patch, represents a clinically relevant but not independently prognostic feature in UC. Across the available comparative literature, AOI appears to be best understood as part of the broader UC disease spectrum rather than a distinct or aggressive disease phenotype.

A key observation from this review is that AOI is most frequently identified in patients with distal or limited disease, and in some cohorts is associated with higher baseline inflammatory activity or reduced rates of complete endoscopic remission [10,12]. This suggests that AOI may reflect localized mucosal immune activation rather than a marker of globally severe disease. Importantly, this distinction helps reconcile the apparent contradiction between increased inflammatory activity and the absence of consistently worse long-term outcomes.

Despite these associations, the overall prognosis of AOI-positive patients does not appear uniformly worse. Multiple studies have demonstrated no consistent increase in hospitalization, colectomy, or other major adverse outcomes [4,6,7,11]. This is a clinically important finding, as it supports a conservative and individualized management approach and argues against escalation of therapy based solely on the presence of AOI.

One of the most consistent signals identified across studies is the potential association between AOI and proximal disease extension, particularly in patients with proctitis or distal UC [8,13]. While this finding suggests that AOI may have some prognostic relevance, it does not translate uniformly into more severe long-term outcomes. Instead, it may indicate a subgroup of patients who warrant closer observation rather than immediate therapeutic escalation.

From a mechanistic perspective, the appendix remains an important and biologically plausible contributor to UC pathogenesis. The appendix is rich in lymphoid tissue and plays a role in mucosal immune regulation and maintenance of the intestinal microbiome. These features provide a potential explanation for why localized inflammation may occur at the appendiceal orifice even in the setting of otherwise distal disease. Recent interest in appendectomy as a potential therapeutic strategy further supports the concept that the appendix may influence disease behavior, although this remains investigational and should not yet guide routine clinical practice [3].

The heterogeneity of findings across studies highlights several important limitations in the current evidence base. Most available studies are retrospective, single-center cohorts with modest sample sizes, which limits generalizability. In addition, there is significant variability in terminology, with studies using AOI, PARP, and cecal patch interchangeably. Outcome definitions are also inconsistent, with different studies focusing on remission, relapse, proximal extension, or treatment escalation. This heterogeneity not only complicates interpretation but also precludes robust quantitative synthesis.

Notably, case reports and small series have also described appendiceal involvement and its potential response to therapy; however, these were not included in the formal analysis due to lack of comparator groups [14,15].

Limitations

This review is limited by reliance on observational studies and by variability in outcome definitions across the literature.

Some older articles and abstracted sources did not provide fully extractable group-level event counts for all endpoints, preventing defensible pooled effect estimates.

Because the evidence base is geographically concentrated in East Asia and a limited number of Western centers, external generalizability should be interpreted cautiously. In addition, although a structured qualitative assessment of risk of bias was performed, the absence of standardized scoring and reliance on observational data should be considered when interpreting the findings.

Another important limitation is the evolution of treatment strategies over time. Earlier cohorts were conducted prior to the widespread use of biologic therapies and treat-to-target strategies, whereas more recent studies incorporate modern endoscopic endpoints such as mucosal healing. These differences may partially explain the variability in reported outcomes and highlight the need for contemporary prospective data.

From a clinical standpoint, the implications of these findings are balanced and pragmatic. AOI should be recognized and documented during colonoscopy, particularly in patients with distal UC, as it may provide insight into disease behavior. However, current evidence does not support using AOI as an independent determinant of treatment strategy. Instead, management decisions should be guided by the overall clinical context, including symptom burden, inflammatory markers, endoscopic severity, histologic findings when available, and longitudinal disease course.

## Conclusions

AOI/PARP/cecal patch is a recognized skip lesion in UC that appears most commonly in limited disease. The current comparative literature suggests possible associations with higher inflammatory activity and proximal extension, but not a uniform increase in major adverse long-term outcomes such as hospitalization or colectomy. AOI should be documented and contextualized, but it should not independently drive treatment decisions.
